# Profil national des indicateurs d’alerte précoce de la pharmaco-résistance du VIH au Cameroun

**DOI:** 10.11604/pamj.2020.37.374.17649

**Published:** 2020-12-23

**Authors:** Serge Clotaire Billong, Joseph Fokam, Calixte Ida Penda, Ernest Anaba Mvilongo, Raoul Fodjo, Arlette Messeh, Jean de Dieu Anoubissie, Cyprien Kegne, Gildas Nguekam, Yannick Aimé Batamack, Edson Joan Billong, Pamela Reine Moutapam, Alexis Ndjolo, Leonard Bonono, Jean Bosco Elat, Anne Cécilé Zoung-Kanyi Bissek

**Affiliations:** 1 Département de Santé Publique, Faculté de Médecine et des Sciences Biomédicales, Université de Yaoundé I, Yaoundé, Cameroun,; 2 Groupe Technique Central, Comité Nationale de Lutte Contre le SIDA, Yaoundé, Cameroun,; 3 Groupe de Travail National pour la Prévention et la Surveillance de la Pharmaco-résistance du VIH, Ministère de la Santé Publique, Yaoundé, Cameroun,; 4 Centre International de Référence Chantal BIYA pour la Recherche sur la Prévention et la Prise en Charge du VIH/SIDA, Yaoundé, Cameroun,; 5 Faculty of Health Sciences, University of Buea, Buea, Cameroon,; 6 Hôpital de Jour, Hôpital Laquintinie de Douala, Douala, Cameroun,; 7 Faculté de Médecine et des Sciences Pharmaceutiques, Université de Douala, Douala, Cameroun,; 8 Division de la Recherche Opérationnelle en Santé, Ministère de la Santé Publique, Yaoundé, Cameroun,; 9 Faculté de Médecine, Université d’Antananarivo, Antananarivo, Madagascar,; 10 George Town University, Yaoundé, Cameroun

**Keywords:** Indicateurs d’alerte précoce, pharmaco-résistance du VIH, Cameroun, Early warning indicators, HIV drug resistance, Cameroon

## Abstract

**Introduction:**

depuis le lancement en 2015 de la stratégie « Traitement pour tous » au Cameroun, un plan d´accélération du traitement antirétroviral (TARV) est mis en œuvre avec des progrès remarquables. Ces efforts s´accompagnent d´un risque d´émergence de la pharmaco-résistance du VIH. L´Organisation Mondiale de la Santé (OMS) propose ainsi la surveillance des indicateurs d´alerte précoce (IAP) de la pharmaco-résistance du VIH. L'objectif de cette étude était d'évaluer sur le plan national les IAP de la pharmaco-résistance du VIH au Cameroun.

**Méthodes:**

une étude rétrospective a été menée en décembre 2017 dans les 10 régions du Cameroun; elle a évalué les six IAPs recommandés par l´OMS sur 68 sites de prise en charge du VIH sélectionnés de façon aléatoire. La période de rapportage s´étendait de Juillet 2016 à Juin 2017.

**Résultats:**

les scores à l´échelle nationale étaient: retrait des médicaments dans les délais (IAP1): 66%; rétention sous TARV 12 mois après l´initiation (IAP2): 66%; rupture de stocks d´ARVs sur une période de 12 mois (IAP3): 53%; couverture en réalisation des tests de charge virale (CV) (IAP4): 10%; suppression de la CV à 12 mois de TARV (IAP5): 73% et pratiques de dispensation du TARV (IAP6): 100%. Les scores régionaux étaient similaires.

**Conclusion:**

la performance des IAP au Cameroun est faible et nécessite des interventions urgentes, prioritairement la couverture des tests de CV, la gestion optimale des ARV et l´adhérence des patients.

## Abstract (en)

**Introduction:**

since the launch of the “Treatment for All in Cameroon” strategy in 2015, an acceleration plan for antiretroviral (ARV) therapy in Cameroon was implemented, with remarkable progresses. These efforts were accompanied by the risk of developing HIV drug resistance. Then, the World Health Organization (WHO) proposed surveillance of early warning indicators (IAP) for HIV drug resistance. The purpose of this study was to assess the national HIV Drug Resistance Early Warning Indicators (EWI) in Cameroon.

**Methods:**

we conducted a retrospective study in the ten regions of Cameroon in December 2017 to evaluate the six EWIs recommended by the WHO in 68 randomly selected HIV care sites. The reporting period ranged from July 2016 to June 2017.

**Results:**

national scores were: drug withdrawal within the estimated time frame (EWI1): 66%; retention on antiretroviral therapy 12 months after treatment initiation (EWI2): 66%; stock-out of antiretroviral drugs over a 12-month period (EWI3): 53%; viral load testing coverage (CV) (EWI4): 10%; coverage suppression after 12 months of antiretroviral therapy (EWI5): 73% and practices for ARV drug delivery (EWI6): (100%). Regional scores were similar.

**Conclusion:**

the evaluation of EWI in Cameroon is limited and requires urgent interventions, primarily viral load testing coverage, optimal ARVs management and patient´s adherence.

## Introduction

Le Cameroun fait face à une épidémie généralisée de VIH. En 2015, un plan national d´accélération du traitement antirétroviral (TARV) a été développé dans l´objectif de mettre fin à cette épidémie à l´horizon 2030 selon les orientations du Programme commun des Nations Unies sur le VIH/sida (ONUSIDA) [1]. En effet la prise correcte d´un traitement antirétroviral (TARV) par les personnes infectées par le VIH permet de réduire des nouvelles infections et la transmission aux tiers [2,3]. Avec une file active de 253 715 personnes sous TARV, le taux de couverture nationale en TARV en décembre 2017 au Cameroun était de 40% versus 57% environ au plan mondial [4,5]. Depuis le lancement de la stratégie « Tester et Traiter » en Octobre 2015, la couverture du TARV est en croissance continue [1,6]. L´utilisation à grande échelle de médicaments à faible barrière génétique augmente les risques de résistance chez les personnes sous TARV, même si les régimes thérapeutiques et l´observance du TARV sont appropriés. Ceci est dû aux erreurs qui surviennent souvent lors de la réplication du VIH et au taux élevé de mutations du VIH lorsque des antirétroviraux exercent une pression sélective sur des recombinaisons virales [7]. L´accélération de la thérapie ARV s´accompagne donc incidemment de l´augmentation du risque d´émergence des résistances.

Les patients hébergeant un virus résistant au TARV sont moins susceptibles de parvenir à la suppression de la charge virale et ont une probabilité plus élevée d´acquisition de nouvelles mutations de résistance [8]. La pharmaco-résistance du VIH peut ainsi mettre en échec le programme national de thérapie antirétroviral (ARV) et faire perdre tout le bénéfice de l´important investissement en cours. Le Cameroun a entre 2016 et 2017 triplé l´approvisionnement en ARV [9] et prévu d´affecter en 2020 près de la moitié de de son investissement dans la lutte contre le VIH [10]. Dans un tel contexte, les stratégies de surveillance de la résistance du VIH aux ARV sont indispensables. Depuis 2008, 06 enquêtes nationales sur la surveillance des Indicateurs d´alerte précoce de la pharmaco-résistance du VIH (IAP) ont été réalisées. Des enquêtes biologiques de la surveillance des résistances transmises ont été effectuées moins régulièrement. Les facteurs relevés susceptibles de favoriser l´émergence des résistances du VIH aux ARV étaient en majorité: la non-adhérence des patients au TARV marquée par la faible rétention et le retard dans le retrait des ARV en pharmacie et les ruptures de l´approvisionnement en TARV au niveau des sites [11-15]. Différentes actions ont été menées dans le but d´améliorer ces indicateurs péjoratifs notamment: le recrutement massif de conseillers psychosociaux pour l´aide à l´observance en 2016 [10] et la mise en place d´un comité national de quantification des ARV assorti du recrutement d´un pharmacien dans chacune des 10 régions du pays pour assurer un suivi décentralisé de l´approvisionnement des pharmacies hospitalières. Dans le cadre de la nouvelle stratégie nationale de lutte contre le sida et les infections sexuellement transmissibles (IST) qui vise à atteindre en 2020, une suppression virale de 90% chez les patients sous TARV, [16] il était important de connaitre le profil national des indicateurs d´alerte précoce de la pharmaco-résistance du VIH. La prévention des résistances évitables pourrait améliorer l´efficacité et l´efficience du programme national de prise en charge par ARV en réduisant l´impact humain et financier de la prise en charge des cas de résistance [17]. En effet au Cameroun, le coût moyen annuel d´un régime thérapeutique de TARV de deuxième intention est sept à huit fois plus élevé que celui d´un régime thérapeutique de première intention. Cette étude avait pour objectif d´évaluer les facteurs spécifiques du programme de TARV qui peuvent être associés à l´émergence de la pharmaco-résistance du VIH au cours du traitement antirétroviral au Cameroun.

## Méthodes

Une étude descriptive et transversale à visée évaluative a été menée au mois de décembre 2017 dans 68 sites représentatifs de l´ensemble des sites de prise en charge par les ARV du Cameroun. Une extraction des données contenues dans différents outils de suivi des patients (registre de traitement, registre de dispensation, fiche de gestion des stocks de médicaments dans les pharmacies, fiche de RDV, logiciels de suivi, etc.) a permis de recueillir des informations permettant de renseigner les 06 indicateurs d´alerte précoce d´émergence des résistances du VIH aux ARV actuellement recommandés par l´OMS. Plusieurs outils étaient consultés pour obtenir une information complète sur chaque patient. En fonction des IAP, il s´agissait de mener une observation transversale ou un suivi historique et longitudinal d´une cohorte de patients. Dans chaque site d´étude, les patients ayant initié le TARV au cours des 03 mois allant d´avril à juin 2016 étaient consécutivement enrôlés jusqu'à l´atteinte de la taille minimale de l´échantillon définie pour le site de TARV. Chaque patient était ensuite observé pendant 15 mois pour les 06 indicateurs dont les seuils étaient présentés en codes de couleurs selon les recommandations de l´OMS: « rouge= mauvaise ou faible; orange= passable; vert= bonne».

Les indicateurs étaient définis de la manière suivante: (i) IAP1-retrait des médicaments dans les délais (le pourcentage des patients qui ont retiré leurs médicaments ARV avant, le jour même ou au plus 2 jours après la date fixée pour le rendez-vous après le retrait initial). Le seuil de la cible était: rouge: <80%; orange: 80-90%; vert: >90%. Précisément les patients qui ont retiré leurs médicaments ARV au mois de février 2017 choisi comme référence ont été sélectionnés. Ensuite, a été enregistré le nombre total de patients (anciens et nouveaux) ayant pris le traitement au cours de ce mois, puis la date du prochain retrait (retrait 1) ou renseigné le cas échéant la date de transfert dans un autre site ou la date de décès, la date d´arrêt de traitement sans reprise après ce mois de référence. (ii) IAP2 - rétention sous TARV à 12 mois après l´initiation au traitement; cet indicateur de type longitudinal évaluait le pourcentage des patients encore vivants et sous TARV 12 mois après l´initiation du traitement avec pour seuil de la cible: rouge <75% de rétention après 12 mois de TARV; orange: 75-85% de rétention après 12 mois de TARV; vert: >85% de rétention après 12 mois de TARV. (iii) IAP3 - rupture de stocks de médicaments; cet indicateur renseignait sur le pourcentage du nombre de mois au cours d´une année donnée pendant laquelle aucune rupture de médicaments ARV n´a été observée. Le seuil de la cible était: rouge: >0% sur une période de 12 mois; vert: 0% sur une période de 12 mois. Il s´agissait de déterminer par une observation transversale le pourcentage de mois sans rupture de stocks dans un site sur la période de 12 mois allant de Juillet 2016 à Juin 2017. (iv) IAP4 - couverture de réalisation des charges virales avec rendu des résultats; cet indicateur a étudié de manière transversale le pourcentage de patients éligibles à l´examen de la charge virale ayant un résultat disponible au niveau des sites à 12 ± 3 mois selon les recommandations nationales. Le seuil de la cible était le suivant : rouge: <70% des patients n´ayant pas une charge virale disponible à 12 mois; vert: >70% des patients ayant une charge virale disponible à 12 mois. (v) IAP5 - suppression de la charge virale à 12 mois de TARV: indicateur de type transversal qui renseignait sur le pourcentage des patients qui ont une suppression de la charge virale (<1000 copies/ml), parmi ceux qui ont réalisé la charge virale à 12 ± 3 mois après initiation du TARV. Le seuil de la cible était: rouge: <75% de suppression charge virale à 12 mois de TARV; orange: 75-90% de suppression charge virale à 12 mois de TARV vert: >90% de suppression charge virale à 12 mois de TARV. (vi) L´IAP6 - pratiques de prescription à l´initiation du traitement ARV: évaluation transversale du pourcentage de patients ayant débuté le TARV sur le site au cours de la période sélectionnée avec prescription d´un protocole conforme aux directives nationales lors de l´initiation du traitement ARV. Le seuil de la cible était fixé à 100%. Pour les IAP2, 4, 5 et 6, la même cohorte des patients a servi de base pour l´évaluation [17]. Pour l´échantillonnage, les 10 régions du pays ont été subdivisées en zone urbaine et rurale, 20 strates (dont 10 urbaines et 10 rurales) ont été systématiquement constituées. Dans chaque localisation géographique régionale urbaine ou rurale, les sites ont été sélectionnés suivant une méthode aléatoire à probabilité proportionnelle à la taille de chaque strate. Sur un total de 379 sites offrant le TARV au Cameroun, un échantillon de 68 (18,2%) sites de TARV a été sélectionné (Annexe 1). La taille des échantillons par site a été déterminée conformément aux recommandations de l´OMS basées sur la formule suivante: N=3.48*p*(1-p)/e^2^. N étant la taille minimale par site à un intervalle de confiance de 95% ± 7% si la proportion réelle des patients atteignant la cible pour l´indicateur est de 50% ; e=précision=0.07. Sur la base de la proportion nationale de la couverture de TARV au Cameroun en fin juin 2017 [18], la taille de l´échantillon d´enfants était proportionnelle au 1/20 de la taille de l´échantillon des adultes.

**Assurance qualité et analyses**: les enquêteurs ont été formés au préalable à l´utilisation des outils et procédures standards de l´OMS dans le domaine de la surveillance des IAP ainsi que sur la gestion globale de l´étude. Conformément aux Normes standards de l´OMS, la validation des données se faisait après un contrôle selon une méthode aléatoire simple sur un échantillon correspondant à 10% de la taille d´échantillon retenu pour le site. Une seule discordance était suffisante pour que les données soient invalidées pour l´IAP concerné et l´extraction reprise. Le programme « Excel » automatisé, Routine Data Quality Assessment (RDQA), développé par l´OMS a été utilisé pour la saisie et l´analyse des données [17]. En outre, une grille de description du site permettait la collecte d´informations complémentaires pour une meilleure compréhension des résultats obtenus localement.

**Considérations éthiques**: cette étude a été réalisée dans le cadre des activités de routine d´évaluation des performances du Programme VIH/SIDA après l'autorisation administrative du Ministère de la Santé. Les patients n´ont pas été interrogé et les enquêteurs étaient des praticiens en charge des données statistiques de routine du programme national de lutte contre le sida. L´étude était basée sur des données historiques, les noms des patients n´étaient pas relevés et seuls leurs numéros d´anonymat étaient enregistrés. Globalement les malades bénéficieront à la suite de cette étude d´une amélioration de leur prise en charge médicale.

## Résultats

Le profil des IAP est présenté selon une codification de couleurs. Soixante-huit sites ont été sélectionnés de manière aléatoire suivant leur poids dans les dix régions : Adamaoua (5), Centre (12), Est (6), Extrême nord (5), Littoral (15), Nord (5), Nord-Ouest (5), Ouest (5), Sud (5), Sud-Ouest (5), parmi lesquels nous avions dont 51 sites urbains et 17 ruraux. On dénombre 07 formations sanitaires privées, 02 hôpitaux militaires, 15 associatives, 06 confessionnelles et 50 formations sanitaires publiques. Sur les 68 sites, plus de la moitié 39 (57,3%) prenaient en charge des patients de 2^e^ ligne, et 5 (7,3%) sites seulement prenaient en charge les patients de 3^e^ ligne. Vingt-huit (28) sites prescrivaient le test de résistance aux patients et enfin seuls quatre sites ne disposaient pas de directives nationales de TARV. Le Tableau 1 ci-dessous présente les résultats de la performance des IAP au niveau national, en signaux de couleurs comme recommandés par l´OMS dans les différents sites de prise en charge (PEC). A l´exception des pratiques de prescription qui sont excellentes dans toutes les régions du pays avec une conformité aux directives nationales de 100%, tous les autres indicateurs d´alertes de la pharmaco-résistance sont au signal rouge (Tableau 1). Une analyse plus détaillée par région permet de décliner les résultats suivants :

**Tableau 1:** niveau d´atteinte des IAP sur le plan national

Classification			Résultats			
Bonne (verte)	Moyenne (orange)	Mauvaise (rouge)	IAP No	Intitulé de l’IAP	Performance	
**>90%**	80-90%	<80%	**IAP1**	Retrait des médicaments dans les délais	**Rouge**	**66%**
**>85%**	75-85%	<75%	**IAP2**	Rétention sous traitement	**Rouge**	**66%**
**0%**	NA	>0%	**IAP3**	Ruptures de stock de médicaments	**Rouge**	**53%**
**>70%**	NA	<70%	**IAP4**	Couverture en charge virale	**Rouge**	**10%**
**>90%**	75-90%	<75%	**IAP5**	Suppression de la charge virale	**Rouge**	**73%**
**100%**	NA	<100%	**IAP6**	Pratiques en matière de prescription	**Vert**	**100%**

**Figure 1:**
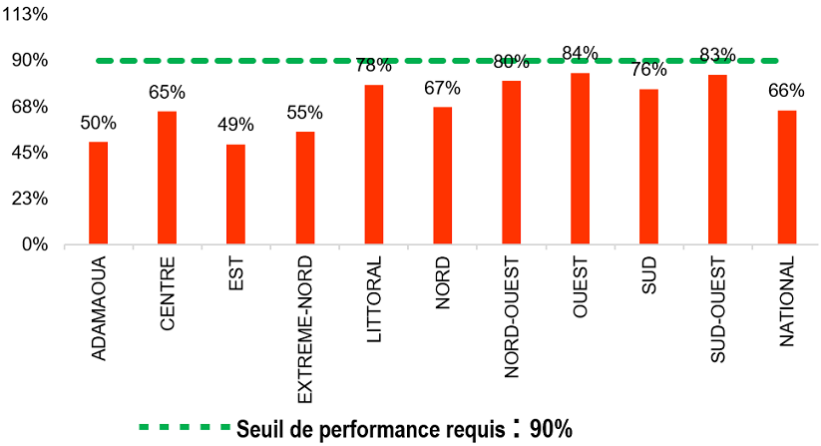
taux de retrait des médicaments ARV dans les délais, file active février 2017 au niveau national (rouge: <80%, orange: 80-90%; vert: >90%)

**Taux de retrait des médicaments ARV dans les délais**: la performance moyenne nationale est de 66%. Aucune Région n´a atteint le taux de performance minimal requis (>90%). Les taux de retrait de médicaments les plus élevés ont été observés dans les régions de l´Ouest (84%) et du Sud-Ouest (83%). Les régions de l´Est (49%) et l´Adamaoua (50%) présentaient les performances les plus faibles; seulement 7 sites sur 68 soit (10,3%) sont au signal vert pour cet indicateur. Aucun site rural (0/17) n´a eu la performance minimale requise. A l´échelle individuelle sur 85.314 patients suivis, 61.049 (71,6%) ont retirés leurs médicaments dans les délais (Figure 1).

**Figure 2:**
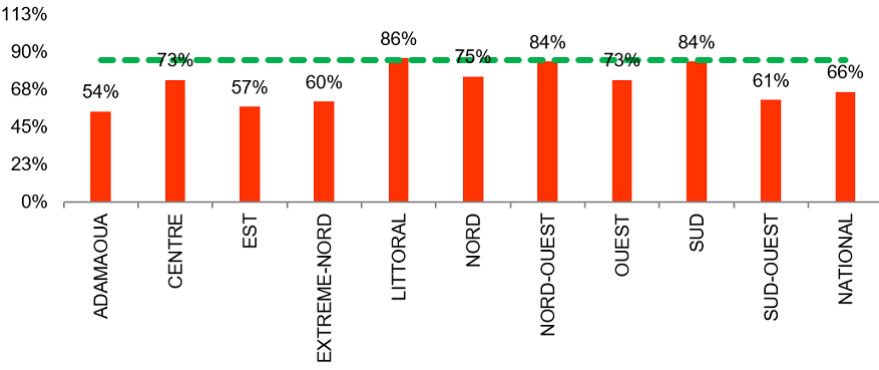
taux de rétention des patients sous ARV au 12^e^ mois de traitement parmi les patients recrutés entre mai 2016 et juillet 2016 au niveau national (rouge: <75%, orange: 75-85%, vert: >85%)

**Taux de rétention des patients sous ARV au 12^è^ mois de traitement**: pour les patients recrutés entre mai 2016 et juillet 2016, le taux de rétention moyen national des patients sous TAR était de 66% pour un seuil minimal de rétention requis >85%. Quatre régions ont eu des performances bonnes à passable comprises entre 75 et 85%: la région du Littoral (86%), la région du Nord (75%), la région du Nord-Ouest (84%) et Sud (84%). Les régions de l´Adamaoua (54%), de l´Est (57%), de l´extrême-Nord (60%), et du Sud-Ouest (61%) ont les plus faibles performances de rétention des malades. Au total 20 (29,4%) sites de prise en charge sur 68 avaient un bon taux de rétention des patients sous TARV, dont 47% (8/17) dans les sites ruraux et 23,5% (12/51) dans les sites urbains. A l´échelle individuelle sur 85.314 patients qui ont initié le TARV en avril 2016, environ 60.943 (71,4%) étaient encore sous TARV (Figure 2).

**Figure 3:**
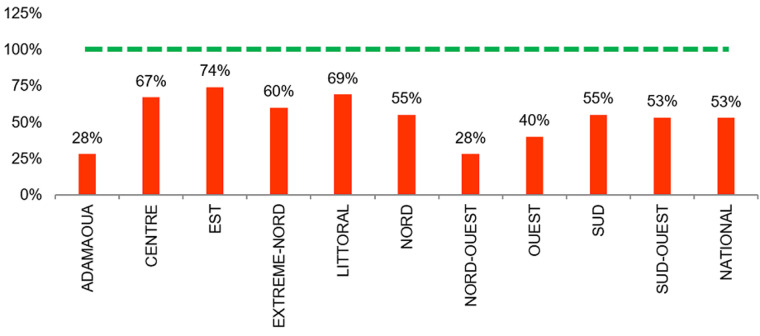
rupture de stock en médicaments ARV au niveau national (rouge: <100%; vert: =100% de mois sans rupture de stock)

**Rupture de stock en médicaments ARV**: le taux de rupture de stock en TARV au niveau national était de 53%. Aucune région n´a connu de mois sans rupture de stocks en TARV. Les régions les plus touchées par la rupture en médicament ARV étaient les régions de l´Adamaoua et du Nord-Ouest avec chacune un score de 28%. Par ailleurs 36 (53,7%) des structures de prise en charge étudiées n´ont enregistré aucune rupture de stock en TAR pendant 12 mois (Figure 3).

**Figure 4:**
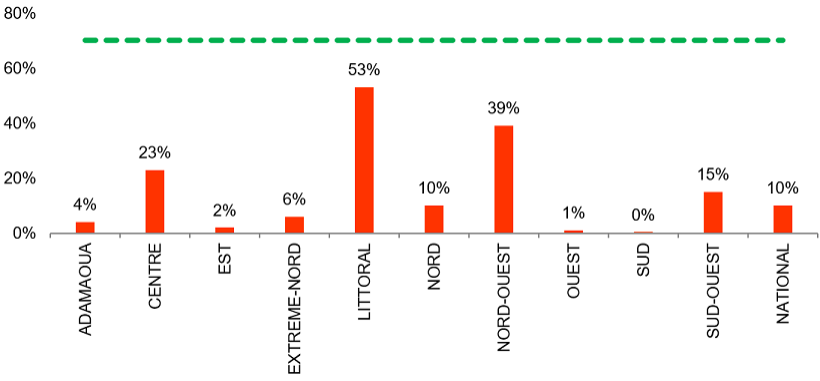
couverture de réalisation des charges virales avec rendu des résultats au niveau national (rouge <70%, vert >70%)

**Couverture de réalisation de la charge virale (CV) avec rendu des résultats**: la couverture nationale était très faible à 10%. Seules les régions du Littoral, du Nord-Ouest, du Centre et du Sud-Ouest ont un taux de réalisation de charge virale au-dessus de la moyenne nationale. La région du littoral (53%) dépassait le seuil de 50% mais elles n´atteignaient pas le minimum requis. Par ailleurs, 7 sites (10 %) sur les 68 sites investigués avaient un taux de couverture nulle en réalisation de CV (Figure 4).

**Figure 5:**
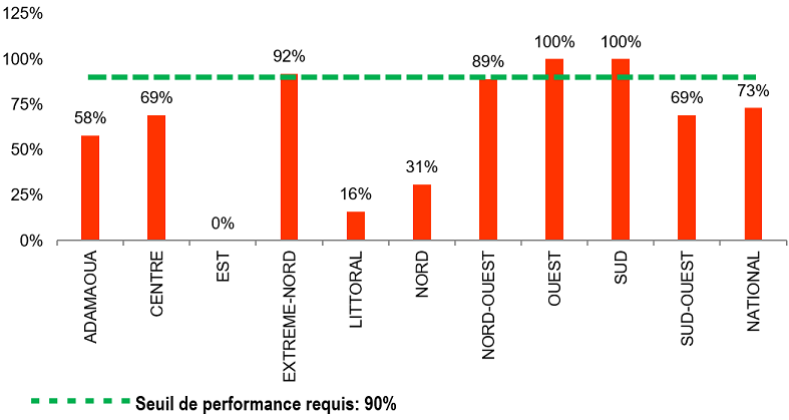
suppression de la charge virale (rouge: <75%, orange: 75-90%, vert: >90%) des patients ayant une charge virale disponible à 12 mois au niveau national

**Suppression de la charge virale des patients ayant une charge virale disponible à 12 mois**: à l´échelle nationale, la suppression de la charge virale des patients ayant une CV disponible à 12 mois était faible car seulement 73% des patients ayant réalisé la charge virale avaient eu une suppression de CV. Toutefois, 3 régions Extrême-Nord (92%), Ouest (100%) et Sud (100%) ont eu de bons scores et avaient dépassé largement le seuil de performance requis. Le Nord-Ouest (89%) a eu un score passable. Par contre les régions du Littoral (18%) et celle du Nord (31%) ont enregistré des faibles scores. En ce qui concerne les sites de prise en charge, 32% (22/68) avaient des scores de suppression de la CV satisfaisant au-dessus de 90%, chez les patients ayant réalisé l´examen à 12 mois (Figure 5). Les résultats détaillés de la performance des IAP par site, urbain et rural, sont rapportés en Annexe 2.

## Discussion

Les IAP explorent les facteurs liés aux comportements des patients (IAP1 et 2), à l´efficacité programmatique du TARV des sites (IAP3, 4 et 6) et à la vulnérabilité du virus (IAP5).

**Le « retrait des médicaments ARV dans les délais » et la rétention des patients sous ARV:** sont très faibles traduisant ainsi la mauvaise compliance au TARV des patients et pouvant expliquer l´émergence de résistances au Cameroun. L´observation a déjà été effectuée plusieurs fois au Cameroun [11,14,15]. Cette situation ne semble pas avoir été adressée au regard des différents rapports programmatiques antérieures [4-6]. Plusieurs études ont rapporté la même problématique dans différents pays à ressources limitées et l´explication récurrente étaient celle de l´inaccessibilité physique des sites de prise en charge des patients souvent très éloignés et situés en zones rurales [11,14,15,18]. En comparant ces résultats au cours des années, nous avons noté un score constant avec 66% des patients qui ont retiré leurs TARV dans les délais en 2017 contre 67% en 2015. Cette situation serait imputable à la faible pratique de l´éducation thérapeutique des patients dans les sites et aux fréquentes ruptures des médicaments ARV dans la plupart des régions.

Une observation plus affinée des résultats des différents sites laisse clairement apparaître le fait que les sites urbains sont aussi concernés que les sites ruraux par cette contreperformance. Ceci relativise la question de la distance souvent évoquée sauf si au vu de la stigmatisation les patients urbains concernés viendraient essentiellement des zones rurales. La qualité du counseling des malades pourrait alors être le facteur explicatif le plus plausible. La problématique de la rétention des malades a fait l´objet de fortes interventions programmatiques au cours des deux dernières années 2016 et 2017. En effet, 1650 Agents psychosociaux (APS) chargés de l´aide à l´observance ont été recrutés par le Comité National de Lutte contre le Sida dont a bénéficié tous les sites de PEC retenus pour l´étude, soit un investissement important de 2,8 milliards de francs CFA, représentant près de 10% de la dépense globale annuelle de la lutte contre le sida au Cameroun en 2017 [4]. Le faible score obtenu indexe l´efficacité et l´efficience de cette intervention et interroge le fonctionnement des comités de rétention en rapport au suivi des patients sous TARV au niveau des formations sanitaires [4]. Cela suggère que cette stratégie devrait se faire avec les mesures d'accompagnement tel que la large diffusion du guide de l'éducation thérapeutique, le renforcement des capacités des APS et la recherche des perdus de vues. En 2017 le Cameroun a débuté la dispensation communautaire des ARV chez les patients stables dans le but d'améliorer la rétention des patients sous TARV. Cependant cette intervention ne pouvait pas influencer ce résultat du fait que les malades éligibles à cette stratégie ont tous au moins 12 mois de TARV et une CV indétectable [19]. Ils n´entraient ainsi pas dans l´échantillon de l´étude. Le taux de rétention à 12 mois de TARV, reste une préoccupation de plusieurs pays à ressources limitées, il était de 51% en côte d´ivoire en 2011 [18]; de 69,1% dans la région du Centre au Cameroun en 2013 [20] et à 60,4 % sur l´ensemble du pays en 2016 [14]. En Namibie seulement 45% des sites en 2012 avait le score requis pour cet indicateur [21]. Bien que la performance reste très faible au Cameroun, une progression lente a été amorcée de 54% en 2015 à 60,4 % en 2016 et à 66% en 2017 soit une nette amélioration attribuable aux différentes interventions menées même si la cible minimale requise (>85%) reste à atteindre.

L´amélioration de cette performance est corrélée à celle de la continuité d´approvisionnement en intrants. Seule la région du Littoral a enregistré un bon score en 2017 (86%) contre (47%) en 2015. Les régions du Nord-Ouest et du Sud ont également amélioré leur score, passant respectivement de 43% et 49% à 84%. Le renforcement de l´éducation thérapeutique, du suivi clinique et biologique et la recherche active des perdus de vue expliqueraient ce résultat. Au Togo en 2017 la performance était excellente à 91 % (3429/3767) [22]. Toutes les régions avaient atteint et dépassé le score minimal requis de 85% du taux de rétention des patients 12 mois après l´initiation, pourtant les conditions logistiques du pays ne semblent pas très différentes de celles du Cameroun. Cette bonne performance était attribuée à la présence de groupes de soutien dans les formations sanitaires. Il s‘agit d´une intervention moins dispendieuse que celle du recrutement des APS et qui donne des résultats efficaces dans un milieu similaire que le Cameroun; l´expérimentation de cette stratégie pourrait être utile dans le programme camerounais de TARV. Le contexte camerounais présente toutefois certaines particularités à prendre en compte dans les analyses et le développement de nouvelles stratégies de rétention des patients, notamment celle des crises sociopolitiques, une étude spécifique de ces milieux pourra être nécessaire. Essomba *et al*. à Douala au Cameroun ont montré que l´oubli du patient était le plus fréquent des paramètres associés à la non observance [23]. Dans ce cas le succès observé au Togo dans la rétention des patients grâce aux groupes de soutien communautaires qui rappellent régulièrement au patient la prise du TARV peut fonctionner mais cette stratégie ne fonctionnera certainement pas dans les régions où le problème sécuritaire constitue la barrière principale au retrait et au maintien des patients sous TRAV. Les stratégies d´aide à l´observance devraient donc être adaptées en fonction des différents contextes.

Les indicateurs de rupture de stock en médicaments ARV et de couverture de réalisation de charge virale ont enregistrés des scores très faibles. Ils traduisaient les difficultés logistiques et fonctionnelles du programme de prise en charge des patients. En effet le niveau national n´avait enregistré aucune rupture d´ARV alors que toutes les régions en étaient victimes. Cette réalité reflète les dysfonctionnements de la chaine nationale d´approvisionnement. La continuité de l´approvisionnement en ARV reste une préoccupation de plusieurs pays à ressources limitées [18,23]. La plus grande préoccupation pour le Cameroun serait cette baisse progressive de performance malgré le nombre de stratégies mises place.

Dans un contexte où les ruptures de médicaments ARV sont fréquentes dans les formations sanitaires, il est possible de se poser la question si les mauvaises performances observées dans la rétention des malades récemment initiés au traitement n´en sont pas la cause. Dans une étude réalisée en 2017 au Togo [23] cette problématique était déjà mise en question. Dans le cadre d´une autre étude il peut être utile de caractériser ce lien. La faible performance de la chaine d´approvisionnement en ARV et autres intrants pourrait ainsi apparaître comme l´un des principaux déterminants de l´émergence des résistances du VIH aux ARV. En effet c´est aussi elle qui semble être indexée dans la très faible performance de l´IAP4. Une étude récente a en effet mis en relief de très nombreuses ruptures d´intrants de CV au Cameroun en 2017 [24]. Le taux faible de la réalisation de la CV pourrait être imputable à l´absence de la gratuité de l´examen bien qu'il soit fortement subventionné au Cameroun. Plusieurs études antérieures ont rapporté le lien entre la pauvreté et l´infection à VIH [25,26]; le prix de cet examen de 5000 FCFA pourrait rester une barrière importante à la réalisation de la CV au Cameroun. Le coût de cet examen est souvent indexé dans les pays à ressources limitées malgré la recommandation de l´OMS de réaliser cet examen dans le paquet de suivi de routine des malades. Au Togo en 2017, le taux de réalisation de la CV était de 5,2%; seulement 178 sur 3429 PVVIH éligibles avaient pu réaliser cet examen [22]. Dans un tel contexte, la **«suppression de la charge virale» IAP5**, est difficile à apprécier à l´échelle nationale. L´OMS et l´ONUSIDA la considère comme l´élément fondamental de mesure de l´efficacité du programme de PEC des patients VIH mais les praticiens hospitaliers auraient tendance encore à prescrire cet examen seulement dans l´évaluation d´un échec thérapeutique en raison de son coût relativement élevé. Les valeurs obtenues de la suppression virale au niveau national devraient tenir compte du biais de mesure. La suppression virale nationale a été mieux évaluée au Cameroun dans le cadre d´une étude plus spécifique en 2017: l´étude CAMPHIA la situait à 80% [27]. Les différentes enquêtes de surveillance ont toujours rapporté de **«bonnes pratiques de la prescription des ARV»** au Cameroun. Cette bonne pratique serait attribuable à la présence de comités thérapeutiques dans les formations sanitaires qui prennent en charge les patients VIH et au succès de la décentralisation. En 2018, dans le cadre de l´accélération de la mise des patients sous TARV, le Cameroun a adopté la directive nationale de «tester et de traiter tous». Cette directive n´intègre pas l´exigence d´un comité thérapeutique malgré l'institutionnalisation de la délégation des taches. Ce nouveau contexte nécessite une surveillance étroite des prescriptions. En effet une régression est possible si l´on prend en compte que certains pays de même niveau de développement ont de faibles performances sur cet indicateur [18].

## Conclusion

Au Cameroun à l´exception des pratiques de dispensation en ARV qui restent excellentes, presque tous les indicateurs d´alerte de l´émergence de la pharmaco-résistance du VIH sont au signal rouge. Des mesures urgentes de prévention de la résistance du VIH aux ARV s´imposent. Notamment la disponibilité continue des ARV et l´accès à l´examen de la CV. Des stratégies innovantes de rétention des patients au programme de TARV nécessitent d´être développées tenant compte des contextes socio-culturels et politiques des régions. L´action des accompagnateurs psycho-sociaux (APS) semble peu efficace, suggérant ainsi d´explorer également le rôle des comités locaux de rétention et l´implication communautaire dans la rétention.

### Etat des connaissances sur le sujet

- L´étude de faisabilité sur l´évaluation des indicateurs d´alerte précoce de la pharmaco-résistance du VIH au Cameroun a été faite sur un échantillonnage dans deux régions du Cameroun ;
- Cinq indicateurs d´alerte précoce de la pharmaco-résistance du VIH avaient été évalués durant cette précédente étude.

### Contribution de notre étude à la connaissance

- La présente étude est une évaluation des indicateurs d´alerte précoce de la pharmaco-résistance du VIH dans les dix régions, sur un échantillon représentatif de la situation nationale;
- Avec le passage dans l´ère du « test and treat » ou « traitement pour tous », cette étude met à jour les informations sur la surveillance des facteurs favorisant l´émergence de la pharmaco-résistance du VIH dans le programme de traitement antirétroviral au Cameroun;
- L´évaluation de la couverture en charge virale est un nouvel indicateur nouvellement considéré dans de telles études au Cameroun, ce qui permet d´évaluer la qualité du monitoring de référence pour en tirer des leçons programmatiques pour ce pays à ressources limitées.

## Remerciements

Nous remercions les responsables des sites de prise en charge qui ont participé activement à la mise en œuvre de cette étude. L´étude a entièrement été financée par le Fonds Mondial de Lutte contre le VIH, la Tuberculose et le Paludisme avec l´appui technique du DAT-AOC de l´ONUSIDA, nous adressons à ces institutions nos sincères remerciements.

## Annexes

**Annexe 1**: présentation de la file active dans les sites sélectionnés

**Annexe 2**: présentation de la cartographie des IAP par site
